# Recent Trends in *S. aureus* and *E. coli*-Based Endometritis, and the Therapeutic Evaluation of Sodium Alginate-Based Antibiotics and Nanoparticles

**DOI:** 10.3390/gels9120955

**Published:** 2023-12-05

**Authors:** Muzammil Talib, Muhammad Ashir Nabeel, Shahbaz Ul Haq, Muhammad Salman Waqas, Huma Jamil, Amjad Islam Aqib, Afshan Muneer, Dalia Fouad, Farid Shokry Ataya

**Affiliations:** 1 Department of Pharmacology, Shantou University Medical College, Shantou 515041, China; dr.muzammil.kamboh@gmail.com; 2 Department of Theriogenology, University of Agriculture, Faisalabad 38000, Pakistan; ashir123nabeel@gmail.com (M.A.N.); vet.master1070@gmail.com (M.S.W.); drhjamil@uaf.edu.pk (H.J.); 3 Department of Medicine, Cholistan University of Veterinary and Animal Sciences, Bahawalpur 63100, Pakistan; 4 Department of Zoology, Cholistan University of Veterinary and Animal Sciences, Bahawalpur 63100, Pakistan; afshanchudhary9@gmail.com; 5 Department of Zoology, College of Science, King Saud University, P.O. Box 22452, Riyadh 11495, Saudi Arabia; dibrahim@ksu.edu.sa; 6 Department of Biochemistry, College of Science, King Saud University, P.O. Box 2455, Riyadh 11451, Saudi Arabia; fataya@ksu.edu.sa

**Keywords:** *S. aureus*, *E. coli*, endometritis, antibiotic resistance, sodium alginate gel, MgO nanoparticles, antibacterial activity, safety

## Abstract

Postpartum infection of the uterus by pathogenic bacteria is exacerbated due to a lack of sufficient epidemiological studies and evidence-based therapeutics. Therefore, this study was planned to find the prevalence, risk factors, and drug-resistance profile of *S. aureus* and *E. coli* isolated from bovine endometritis and to evaluate the antibacterial potential of sodium alginate-based antibiotics and nanoparticles. The study revealed 34.21% *S. aureus* and 31.57% *E. coli,* whereas most of the assumed risk factors presented significant association in this study. *S. aureus* showed the highest resistance against fusidic acid (60%) and cefoxitin (50%), while the highest resistance in *E. coli* was found against fusidic acid (60%), gentamicin (60%), chloramphenicol (50%), and cefoxitin (50%). Tylosin coupled with MgO nanoparticles stabilized in sodium alginate gel (Tylo + MgO + gel) presented significantly lower minimum inhibitory concentration (MIC) against *E. coli*, showing 13.88 ± 4.51 µg/mL after 24 h incubation. On the other hand, gel-based preparations showed MIC as 31.25 ± 0 µg/mL (Tylo + gel + MgO) and 26.04 ± 9.02 µg/mL (Tylo + Gel) against *S. aureus*. Generally, the MICs of non-gel-based preparations were significantly higher against bacteria except ampicillin against *S. aureus* in this study. The toxicity analysis of MgO nanoparticles presented 20–80% mortality of snails against a wider range of 0.01 mg/mL–10 mg/mL. The histopathological parameters concluded MgO nanoparticles safe to use on off targets. The current study thus concludes the rise in antimicrobial resistance while the gel-based products appearing as effective antimicrobials with sufficient safety margins for off-targets. The study thus invites further investigation for the development of suitable and affordable modified therapeutics for better health and production of animals.

## 1. Introduction

Reproductive diseases such as pyometra, metritis, endometritis, retained fetal membranes, and general uterine infections affect the fertility of dairy animals [1]. Among dairy cows, endometritis and metritis contribute to infertility, lower performance, and early culling. Purulent or mucopurulent discharge from the vaginal area after delivery (3 weeks or later) or after giving birth is clinical endometritis. Another definition of subclinical endometritis is the presence of neutrophils in biopsy or histological examination of the endometrium without any clinical symptoms [2]. Endometritis is one of the diseases with the highest prevalence in dairy cows during the early days of the postpartum, shortly after birth [3]. Bacterial etiology includes but is not limited to *Staphylococcus aureus* [4], *Escherichia coli* (*E. coli*), and *Trueperella pyogenes* [5]. Cows suffering from metritis have decreased ovarian functionality [6], and it goes to unleash in the presence of bacterial infections like *S. aureus* and *E. coli*, as these are common pathogens circulating in humans and animals. Alternatives to antimicrobials have been used to tackle the growing issues of antimicrobial resistance [7]. These pathogens remain a serious concern for public health as metritis is often a sequel of mastitis and thus can sufficiently be harmful to humans who deal with animals or consume milk [8]. *S. aureus* itself is expressed as an adaptive pathogen that generates different strains that can compromise dairy health [9]. On similar aspects, *E. coli* stands as a versatile pathogen among Gram-negative pathogens.

Antimicrobial resistance (AMR) is a worldwide phenomenon posing a serious threat to public health [10]. Specifically, the inactivation of binding sites, modification of the target sites, and alteration of the metabolic pathways are salient mechanisms that, alone or in combination, bring resistance against antimicrobials [11]. It is possible to improve antibiotic stewardship by implementing an appropriate treatment and use of alternatives to antimicrobials thus decreasing the morbidity [12]. The livestock industry has already been sufficiently compromised due to COVID-19, especially in developing countries like Pakistan [13]. In such a scenario, the rise in antimicrobial resistance due to a lack of resources becomes an additional burden.

At present, nanotechnology is a key component of medical and pharmaceutical advances. Nanoparticles have a more excellent surface-to-volume ratio, are stable, bioactive, bioavailable, and possess a controlled particle size. Combining nanoparticles with antibiotics, antimicrobial peptides, and essential oils is believed to lessen mammalian cell toxicity. Thus, it is wise to find modified approaches to maximize antibacterial activity with minimal toxicity. A useful approach to extend the shelf life of preparations is stabilization in gel-based compounds. Many biomedical applications use sodium alginate gel, including drug delivery, tissue engineering, food preservation, and nanomedicines. Several previous studies have shown that the gel has antibacterial properties, while its use in wound dressings has also been proven to prevent secondary bacterial infections [14]. Nanomedicine uses gel in various ways, for example, in dendrimers, emulsions, lipids, nanocrystals, and polymeric nanoparticles. These are easily biodegradable with excellent biocompatibility, lower toxicity, and higher antimicrobial activity. Studies on toxicity evaluation of heavy metals in other food animals have been studied [15,16], while the nanoparticles need to be evaluated for these parameters. Thus, current study aimed to find the prevalence of *S. aureus* and *E. coli* from endometritis and to evaluate gel-based antibiotics and nanoparticles as effective and safe drug-resistance modulators.

## 2. Results and Discussion

### 2.1. Characterization of Nanoparticles

#### 2.1.1. XRD Analysis of MgO Nanoparticles

**Figure 1:**
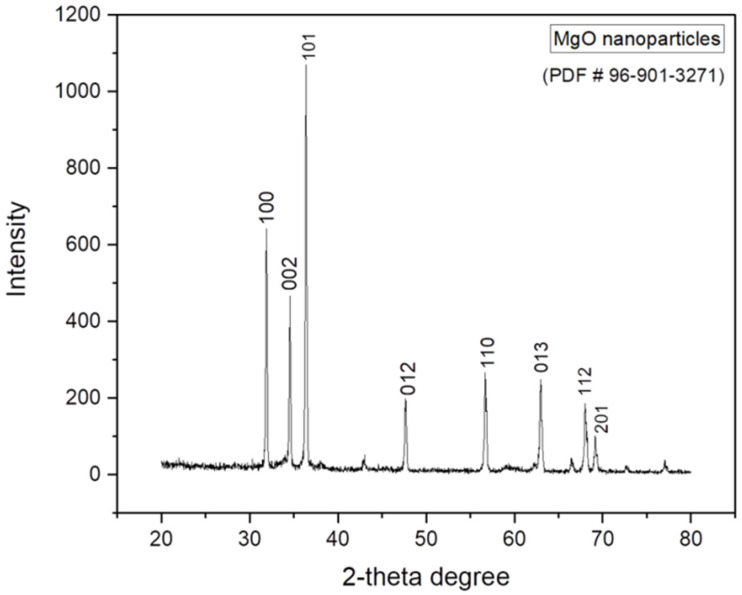
XRD pattern of MgO nanoparticles.

XRD patterns of the synthesized product were plotted between 2-theta (°) values and the intensity which are shown in Figure 1. Millar indices for diffraction peaks at 2 theta (°) 31.80°, 34.50°, 36.30°, 47.60°, 56.60°, 62.91°, 68.03°, and 69.08° are (100), (002), (101), (012), (110), (013), (112), and (201), respectively, (PDF # 96-901-3271) and the JCPDS number (75-1525) was completely matched with that of mentioned in [17]. This set of 2-theta (°) values and corresponding indices shows that the synthesized product is MgO nanoparticles. This lattice has a cubic crystal system (CCS) and lies in the F m -3 m space group with unit cell isa = 3.8280 Å and an intensity scale factor of 0.07. Some peaks were very sharp, indicating that the product is highly crystalline. No extra peak was observed in the XRD pattern of MgO nanoparticles, and this confirmed that the product is highly pure. Very low-intensity peaks were not identified because of the noise. As the peak intensity increases, the particle size decreases due to the material being within the nano range. This present value is in good accordance with the literature reports. 

#### 2.1.2. SEM Analysis

**Figure 2:**
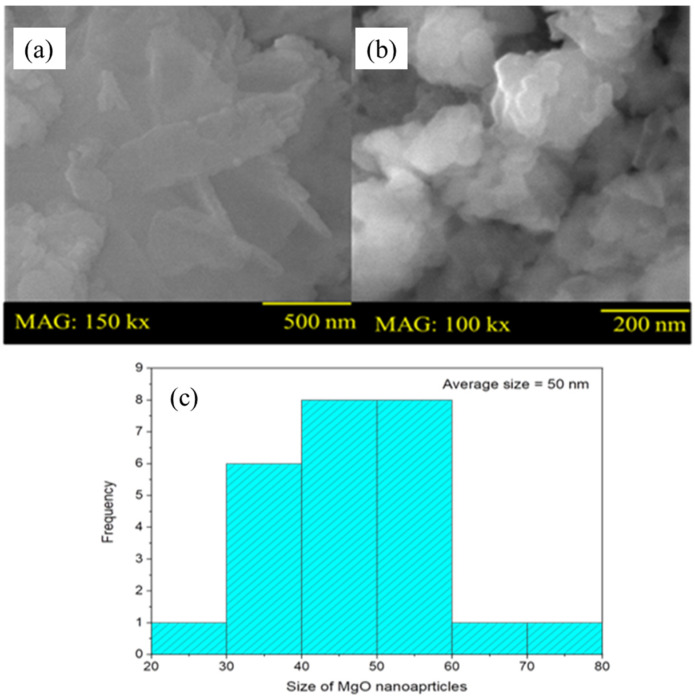
Scanning Electron Microscopic image of MgO nanoparticles (**a**,**b**) and histogram (**c**).

SEM analysis was conducted to acquire information regarding the morphology and size of the synthesized MgO nanoparticles. For this reason, the SEM images of MgO nanoparticles were obtained at different magnifications depicted in Figure 2a,b. In Figure 2a, the morphology of the MgO was observed and revealed that the particles were spheroids with round ends and joined at different points and with smooth surfaces [18]. These results show that some of the particles are dispersed entirely, and some of them are aggregated. The average size of the nanoparticles was 50 nm. In Figure 2b, the synthesized product consists of numerous randomly arranged particles with controlled morphology of MgO. In the literature, the spheroid shapes of MgO exhibit a uniform and rounded morphology, which can benefit various applications due to their specific geometrical characteristics. The size frequency of MgO nanoparticles with spheroid morphology is depicted by the histogram plot in Figure 2c. The average size of the MgO was approximately 50 nm. 

#### 2.1.3. UV–Visible

**Figure 3:**
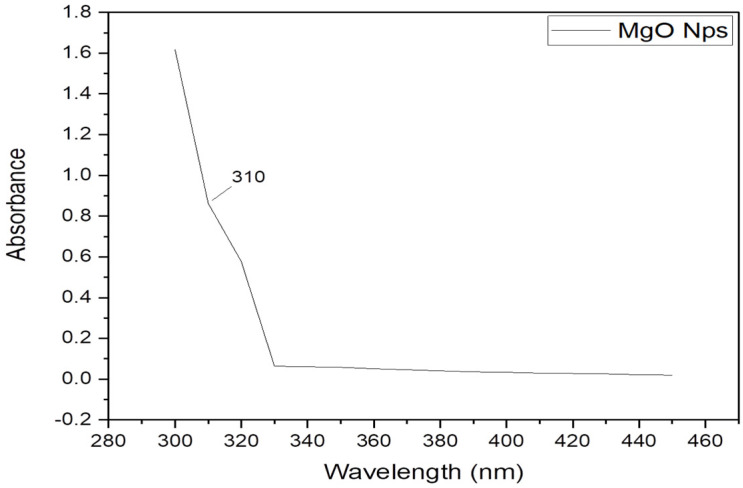
UV–visible spectra of MgO nanoparticles.

Using absorption spectra recorded in the 300 to 450 nm UV–visible wavelength range, the characteristics are displayed in Figure 3 at 310 nm. It shows the absorption because of bulk excitation transition for a single crystal of MgO nanoparticles; this absorption was significantly higher than that of bulk MgO [19]. The low coordination was shown by the high wavelength of the surface excitation associated with the absorption at 310 nm, which was attributed to the coordination of the surface oxide ions. This was because as coordination decreased, the electronic potential of an O^−2^ ion in MgO progressively dropped thus requiring less energy overall.

#### 2.1.4. Raman Analysis

**Figure 4:**
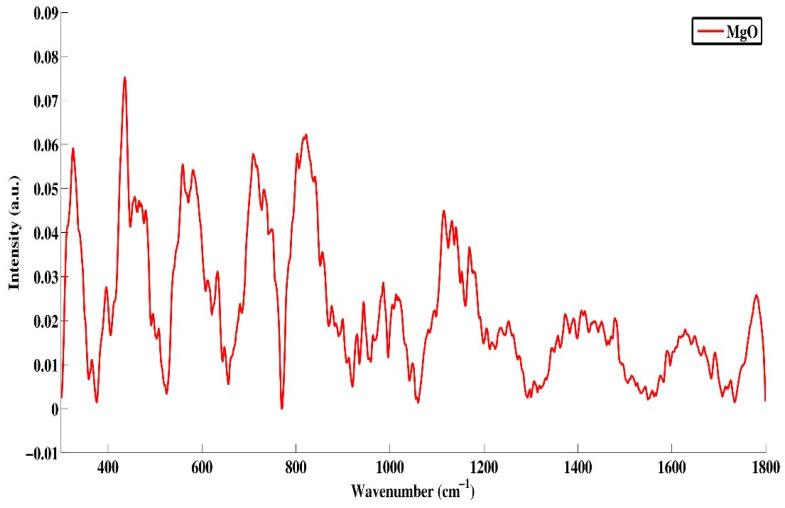
Raman spectra of MgO nanoparticles.

Raman spectroscopy is extensively used to consider the microstructure properties and explore the structural defects of prepared MgO nanoparticles. The response of magnesium in pure magnesium oxide was represented in the spectra by significant peaks that appeared at wavenumbers 270, 390, 460, 580, 650, 980, 1090, 1140, 1340, and 1590 cm^−1^ [20,21] in Figure 4. The peaks at 270 and 460 cm^−1^ were the characterization of the MgO nanoparticles. According to the results, the peak at 270 cm^−1^ was due to the TA phonon at an energy zone boundary, and that of at 460 cm^−1^ was due to the phonon at the energy zone center in MgO nanoparticles. The peak at 650 cm^−1^ was due to a defects-induced mode linked with the lattice flaws, i.e., vacancies in oxygen, magnesium interstitials, or a combination of these elements [22]. Furthermore, the peak at 390 cm^−1^ corresponding to the MgO (LA) stretching mode was very sensitive to oxygen vacancies. They suggested that the peak at 980 cm^−1^ was attributed to the LA + LO vibrational mode in MgO due to the synthesis of the product and the salt effects. The line at 1080 cm^−1^ and the line at 1120 cm^−1^ that made up the vast band were comparable to those previously observed and attributed to the surface phonon modes in a TO–LO phonon gap [23]. Because of the structural changes brought about by disorder and particle size reduction, the increased specific surface area may cause the intensity of vibrational modes to decrease, and as a result, the σs values of such NPs were lower.

### 2.2. Prevalence and Risk Factors of Endometritis S. aureus and E. coli

**Figure 5:**
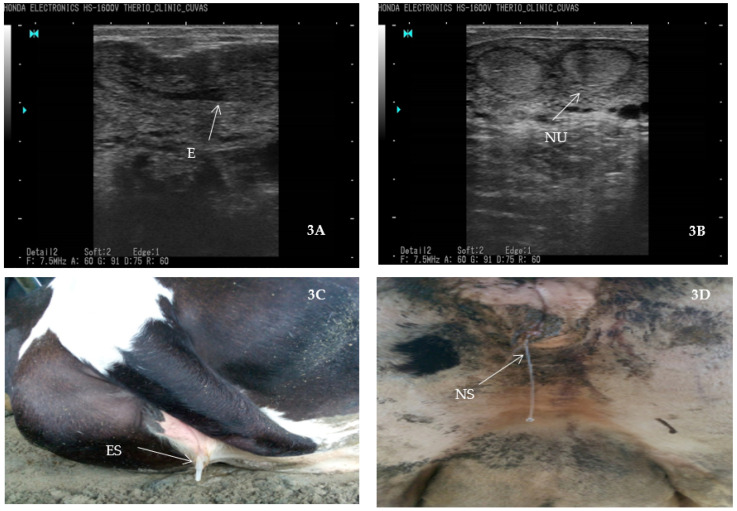
An ultrasound image showing cattle with endometritis. 3A, 3B, and 3C indicate endometritis secretion (ES) from the reproductive tract of animals, which was the basis for inclusion criteria for sample collection; 3D indicates normal secretion from the reproductive tract of animals. NU = normal uterus, E = Endometritis, ES = endometritis secretion, NS = normal secretion.

The current study found 43.75% of animals positive for endometritis whereas the overall prevalence of *S. aureus* and *E. coli* was 34.21% and 31.57%, respectively. The percentage of *S. aureus* from endometritis cases was 78%, while that of *E. coli* stood at 72.18% (Table 1 and Table 2). The clinical picture of endometritis and its confirmation through ultrasound has been depicted in Figure 5.

The prevalence of endometritis in the current study was in line with the findings of Baran et al. [24], who reported 42.4% prevalence of endometritis in their study. Contrary to the findings of the current study, the observations of Gilbert [25], Denis-Robichaud and Dubuc [26], Nyabinwa et al. [27], and Kumar et al. [28] reported 53%, 52.7%, 70.2%, and 72.22% prevalence, respectively. The discrepancies in reporting prevalence in the current study with those of other studies might be because of differences in management practices, detection methods, breeds of animals, immune responses, and climatic conditions. A study on the etiology of endometritis in dairy cows in China found *S. aureus* to be the most common pathogen related to the disorder [4]. In the previous study by Udhayavel et al. [29], the same bacteria as that of the current study were the most prevalent pathogens. Another study reported 83.33% of the samples to be positive for endometritis, whereas *E. coli* accounted for 36.66% of these samples [30]. Despite the findings of the current study, nominal percentage of Japanese Holstein cattle were found to have endometritis, and *E. coli* stood as the most common pathogen [1]. A similar study, in agreement with the current study, reported *E. coli* as the most common bacterium isolated from uterine samples of Holstein cattle [31].

The assumptions of risk factors associated with the spread of *S. aureus* were found to be significantly (*p* < 0.05) associated with calving history, milk yield per day, days in milk, parity, and feeding regime (Table 1). Conversely, the treatment approach did show a non-significant (*p* > 0.05) association with *S. aureus*. Regression analysis for risk factors associated with *S. aureus* showed a parity range (1–3) with 2.983 odds, days in milk (101–200) showed 13.5 odds at *p* < 0.05 and feeding regime (Silage + Concentrate showing) 56 odds at *p* < 0.001. Similarly, several assumed risk factors were significantly associated with the spread of *E. coli* including calving history, milk yield, days in milk, parity, and feeding (Table 2). Regression analysis on assumed risk factors for *E. coli* revealed calving dystocia with 1.538 odds of acquiring an infection, while an abortion had 31.154 odds. Silage + Concentrate had 78.0 odds of acquiring *E. coli* infection.

### 2.3. Antibiotic Susceptibility of S. aureus and E. coli against Antibiotics

The current study showed the highest resistance in *S. aureus* against fusidic acid (60%), followed by cefoxitin (50%), levofloxacin (30%) and linezolid (30%). The highest sensitivity was shown against gentamicin (80%) followed by vancomycin (70%) and ciprofloxacin (70%). The present study showed the highest resistance in *E. coli* against fusidic acid (60%) and gentamicin (60%). In the case of enrofloxacin, trimethoprim-sulfamethoxazole, vancomycin, and ciprofloxacin, 70–80% of *E. coli* isolates were found sensitive (Table 3).

Higher resistance in *E. coli* to these antibiotics could be attributed to the prevailing conditions of overuse and misuse of these medicines in hospitals and clinics. A high resistance to penicillin G and amoxicillin was observed in *E. coli* isolated from cattle, whereas amoxicillin and ciprofloxacin were found to be sensitive [32]. There is important evidence that this pathogen is becoming resistant to chloramphenicol, gentamicin, and oxytetracycline. Furthermore, it was reported previously that livestock-associated *E. coli* isolates were highly resistant to chloramphenicol [33].

### 2.4. Antibacterial Potential of Gel-Based Nanoparticles and Antibiotics

**Figure 6:**
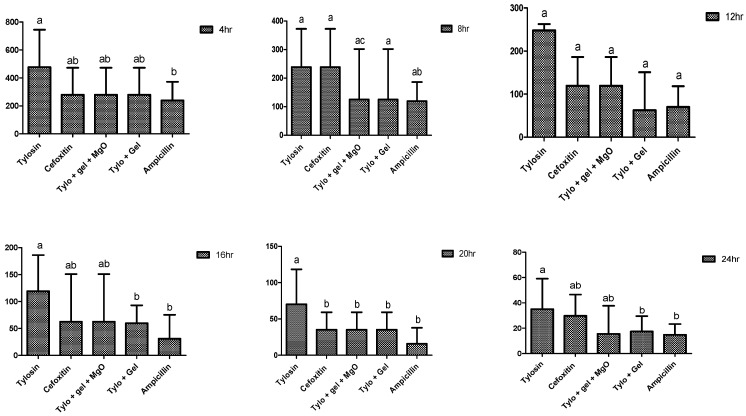
Minimum inhibitory concentration (µg/mL) of different preparations against *S. aureus* at incubation periods (hours). Different alphabetic letters (a, b, c) on bar graphs indicate a significant difference (*p* < 0.05) among treatment groups, hr = hour.

Current study noted that the gel-based preparations had a significant antibacterial potential compared to non-gel-based preparations throughout the study observations (Figure 6). Among different preparations, the lowest minimum inhibitory concentration (MIC) was found in favor of Tylo + gel + MgO, presenting 31.25 ± 0 µg/mL against *S. aureus*. Among the non-gel-based preparations, tylosin (52.08 ± 18.04 µg/mL) and cefoxitin (41.67 ± 18.04 µg/mL) showed the highest MIC after 24 h of incubation against *S. aureus* but ampicillin showed 20.83 ± 9.02 µg/mL which was lesser than that of Tylo + Gel (26.04 ± 9.02 µg/mL).

**Figure 7:**
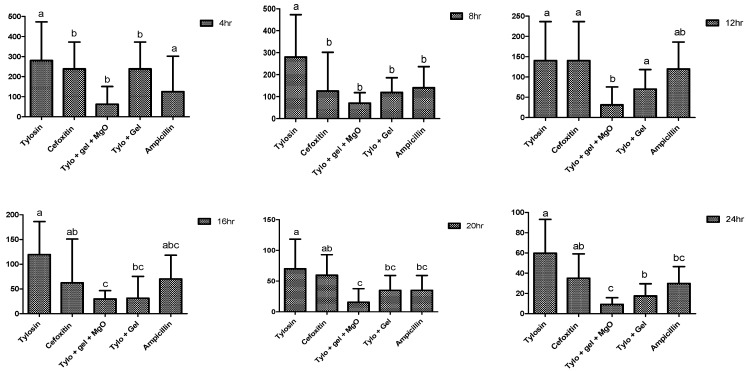
Minimum inhibitory concentration (µg/mL) of different preparations against *E. coli* at incubation periods (hours). Different superscripts indicate significant differences (*p* < 0.05). Different alphabetic letters (a, b, c) on bar graphs indicate a significant difference (*p* < 0.05) among treatment groups, hr = hour.

The antibacterial potential of the gel-based preparations was pronounced among all other preparations against *E. coli* compared to their responses against *S. aureus*. At 4 h of incubation, the gel-based preparations showed decreased MIC compared to the non-gel-based preparations throughout the trial (Figure 7). The lowest MIC at 4 h incubation was noted in Tylo + gel + MgO (125 ± 0 µg/mL), while the highest was found in tylosin (416.66 ± 144.33 µg/mL). Following 24 h of incubation, the lowest MIC was noted in the case of Tylo + gel + MgO, followed by Tylo + gel, ampicillin, cefoxitin, and tylosin, showing 13.88 ± 4.51, 26.041 ± 9.021, 52.08 ± 18.0, and 83.33 ± 36.08 µg/mL, respectively.

Studies have shown that nanoparticles control bacterial infection both in vivo and in vitro by either appearing as an antibacterial or enhancing the antibacterial activity of antibiotics [34]. Research has shown a significant improvement in antibacterial properties and anti-MRSA (methicillin-resistant *S. aureus*) properties when metal nanoparticles were combined with antibiotics as mentioned in the study of Dibrov et al. [35]. Allahverdiyev et al. [36] also demonstrated that combining amoxicillin with silver nanoparticles effectively modulated resistance in *E. coli*. Similar findings were also observed by Li et al. [37] in 2005 against *E. coli*. An earlier study found that superparamagnetic iron oxide nanoparticles (SIONPs) at 5 mg/mL inhibited the growth of Gram-positive and Gram-negative bacteria in biofilms [38]. Researchers found that propolis-treated sodium alginate based products increased zone of inhibition more than 50% compared to the use of nanoparticles alone against bacteria [39]. Metallic oxide nanoparticles, however, were shown to significantly slow down the growth of *Streptococcus* and *Klebsiella* [40].

### 2.5. Histopathology

**Figure 8:**
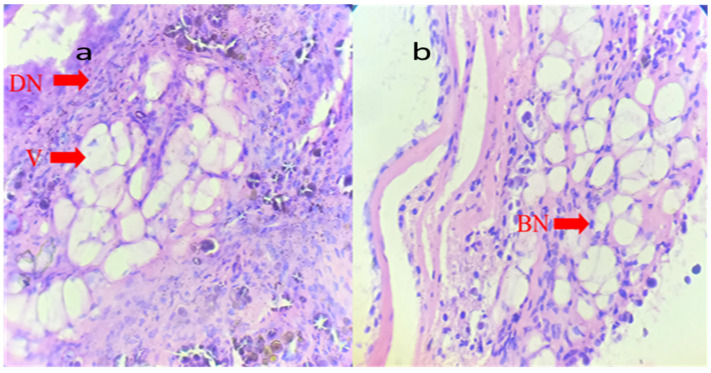
Histopathological expressions on snail digestive glands (stained with H&E) under the effect of sodium alginate-stabilized compounds (**a**) Cells from digestive glands treated with 4.883–26.04 µg/mL sodium alginate-stabilized nanoparticles and antibiotics (at 400×) show vacuolar degeneration with marginal nuclei (red arrows) despite some normal cells. An arrow indicates the central nucleus and the large and light nuclei. (**b**) MIC for 10-fold products (48.83–260.4 µg/mL). The digestive gland treated with sodium alginate-based compound (at 400×) shows vacuolar degeneration (red arrow) and pyknotic nuclei, which indicates cellular death Degradation (red arrow), DN = digestive cell nuclei, V = vacuole, BN = Basophilic nuclei.

Digestive glands were denatured, whereas calcium cells and excretory cells were found converting to excessive vacuolation; granules were released when digestive cells were denatured; basophilic infiltration was visible as a sign of a pathological condition; metallic nanoparticles were found infiltrating at various sites of the digestive glands (Figure 8). Connective tissue was denatured, and the lumen widened, eventually disfiguring the structure. Calcium cells with bigger calcium spherules were packed together resulting in pyknotic nuclei. Some areas with debris had excretory cells visible.

### 2.6. Toxicity Evaluation

The magnesium oxide nanoparticles utilized in this study had minor off-target effects, according to the ecotoxicity investigation using a snail model (Table 4). MgO nanoparticles were shown to have the highest death rate in the starving snails. The concentration of preparations was shown to be directly related to mortality with larger concentrations resulting in increased mortality. On the 5th day of the study, two snails in the control group died. Compared to the first and fifth days of the study, most snails died on the third day.

A snail is an excellent model for assessing the ecotoxicity of nanomaterials since they are bio-accumulators and a part of terrestrial and aquatic ecosystems [16]. A study reported a direct correlation between CuSO_4_ concentrations and the exposure times and tissue lesions that correlated directly with their potential and severity [41]. Nanomaterials have a variety of physical and chemical properties that influence their ability to act as molluscicides against snails and their environmental impact, as mentioned in Caixeta et al. [42]. Biochemical analyses of animal sera showed ZnO nanoparticles to be safer, as concluded from the in vivo trial. Some of other studies also showed similar results after five days of the treatment on the dermis [43,44].

## 3. Conclusions

The present study revealed a high prevalence of *S. aureus* and *E. coli* from bovine endometritis. Except the feeding regimen in case of *E. coli*, all other assumed risk factors showed significant association with the spread of these pathogens. On the other hand, the antibiotic susceptibility of both bacteria against different antibiotics was found to be decreasing. Sodium alginate-based (gel-based) preparations of nanoparticles and antibiotics showed a significant antibacterial potential compared to the non-gel-based preparations against both bacteria; however, the said trend was pronounced against *E. coli*. Among all the preparations, gel based MgO nanoparticles presented the highest antibacterial potential at different hours of incubation. It was also noteworthy that these preparations were also effective in the early hours of incubations and therefore suggest that the gel-based products can tackle sudden or acute infection. The toxicity analysis of MgO nanoparticles showed it to be safe to use. The study thus proposes immediate measures to be taken to tackle antimicrobial resistance using a modified alternative antimicrobial combination like the ones used in this study. Furthermore, in vivo studies along with the exploration of molecular mechanisms are necessary steps to be taken.

## 4. Materials and Methods

### 4.1. Synthesis of Nanoparticles

The basic materials for the synthesis of MgO nanoparticles were Mg (CH_3_COO) 2.4H_2_O and C_2_O_4_.2H_2_O. All these components were dissolved in methanol. A magnetic stirrer was applied continuously to stir and heat approximately 50 g of Mg (CH_3_COO) 2.4H_2_O in 150 mL of methanol. To correct the pH of the resultant solution, 1.0 m of oxalic acid solution was added and stirred until a white gel formed. After the gelation process had begun, it was maintained at the normal room temperature for one night. Whatman-42 filter paper was applied to separate the gel from its solution. To remove water and acetate trapped in the particles, they were heated at 200 °C for 24 h. Furthermore, the dried product was crushed with a mortar and pestle to create a fine powder sieved in a 100-mesh sieve to make a magnesium oxalate complex to prepare the MgO nanoparticles. The complex was produced and annealed at 550 °C temperature and 1 atm for 6 h.

### 4.2. Characterization of Nanoparticles

MgO nanoparticles were characterized by the X-ray diffraction technique method. Coherent scattering domains (CSDs) and micro strains on the lattice were smaller than expected. DRON-4 powder diffractometer (Cuk radiation and pyrolytic graphite monochromator were used for XRD). Polycrystalline silicon (220 reflection, 2 = 47.34°) was used as a peak-shape reference. Using the 200-diffraction line profile (2 = 42.98°), the size of the CSD was calculated in magnesium oxide. The morphology of powder specimens was analyzed using a JEOL 5910 scanning electron microscope (SEM). Using a MOM Q-1500 D thermos analytical system and alundum crucibles, a thermal analysis (DTA + TG + DTG) was performed at a heating rate of 10 °C/min [45].

### 4.3. SEM Analysis

A hydrothermal method was applied to synthesize MgO nanoparticles, followed by calcination. MgO nanoparticles calcined through SEM were studied for morphological characteristics. A surface study of MgO was conducted using SEM images scanned at 150 kx and 100 kx magnifications.

### 4.4. Toxicity Analysis

The toxicity of nanoparticles was tested on *n* = 30 healthy land snails collected from an untreated garden without any pesticides or chemicals. Snails were divided into two groups: a treatment (group 1) and a control (group 2) (Table 4). The treatment group was further divided into four groups (10 mg/mL, 1 mg/mL, 0.1 mg/mL, and 0.01 mg/mL) based on the concentration of MgO nanoparticles provided to the snails. Each concentration of MgO nanoparticle was randomly allocated to *n* = 5 snails, a total of *n* = 20 snails in the treatment group, while that of the control group comprised *n* = 10 snails. The solution of each preparation was poured onto the anterior side of the snail’s mouth portion. Dead snails were immediately processed for histopathology after being kept off feed for 5 days [14].

### 4.5. Histopathology

A snail was dissected, and its digestive glands were collected in Bouin’s fluid. Sections with a thickness of 5 mm were deparaffinized, hydrated, and stained with hematoxylin for 15 min. Xylene was used to clean the cells after alcohol dehydration, then Canada balsam was mounted on them, and thin cover slides were finally used. Pathological changes such as disintegration, abnormal nuclei, karyolysis and severe karyorrhexis, pyknosis, excessive vacuolation, and frequent dark granulations were observed in these cells [14].

### 4.6. Uterine Sample Collection

#### 4.6.1. Tracking Bovine Endometritis

A veterinarian supervised the ultrasonography study conducted on dairy farms with *n* = 50 or more lactating cows suspected of having endometritis [46]. For this study, *n* = 10 commercial dairy farms in southern Punjab, Pakistan, were approached. A convenient sampling technique was used in this study to screen *n* = 304 exotic cows for endometritis. The reproductive tract was visualized using an ultrasound machine (7.5 MHz linear array transrectal probe, B mode), and endometritis was identified partially by echogenic snowy patches in the lumen [47]. A distended uterus with whitish and grey areas indicated that the uterus was inflamed and surrounded by echogenic fluid. When the light passed through a structure, many rays returned that looked hypoechoic (grayscale). The hyperechoic (whitish) appearance is due to the reflection of all rays from any structure [48]. As a result of endometritis, some ultrasound rays passed through pus inside the uterine lumen, and some of them reflected, leading to a mixture of hypo and hyperechoic images. In an artificial insemination procedure, sheath fluid was flushed through the uterus aseptically using a syringe and artificial insemination gun. A cold chain of 4 °C was maintained by shipping samples to the Department of Theriogenology, University of Agriculture, Faisalabad, Pakistan.

#### 4.6.2. Isolation of *S. aureus* and *E. coli*

Samples were incubated in a sterile nutrient broth overnight at 37 °C, followed by centrifugation at 6000 g for 10 min. A sterile swab was dipped in the sediment of this centrifuged material which was subsequently spread over blood agar and put to 24 h of incubation at 37 °C. Mannitol salt agar and MacConkey agar were further used as differential media to isolate *S. aureus* and *E. coli*. Using guidelines in Bergey’s Manual of Determinative Bacteriology, biochemical tests were performed on bacterial growth obtained from differential media. The pooled information was used to confirm particular bacteria focused in this study [49].

#### 4.6.3. Antibiotic Susceptibility

The antibiotic susceptibility profile was carried out using the disc diffusion method as described in Clinical Laboratory and Standard Institute [50]. Antibiotics namely fusidic acid (10 µg), ciprofloxacin (5 µg), amoxicillin (30 µg), vancomycin (30 µg), gentamycin (10 µg), linezolid (30 µg), and cefoxitin (30 µg) were tested. Antibiotic discs were aseptically placed on Mueller–Hinton agar plates following the spread of fresh bacterial culture adjusted at 1–1.5 × 10^8^ CFU/mL. The assembly was put to incubation at 37 °C for 24 h. Zones of inhibition were measured and compared with standards provided in Clinical Laboratory and Standard Institute to declare strains as resistant, intermediate, or sensitive.

### 4.7. Evaluation of Gel Based and Nongel Based Preprations

#### 4.7.1. Well Diffusion Assay

Sterile Mueller–Hinton agar plates were put further to the formation of wells of 6–8 mm diameter using a well borer. Fresh growth of bacteria adjusted at 1–1.5 × 10^8^ CFU/mL was swabbed on Mueller–Hinton agar. Preparations were poured into the wells and put to incubation at 37 °C for 24 h. Zones of inhibition were measured using a vernier caliper following the incubation.

#### 4.7.2. Minimum Inhibitory Concentration

To determine the minimum inhibitory concentration (MIC), the broth microdilution method was carried out. For this, bacterial strains were adjusted at 1 × 10^5^ CFU/mL and were poured in all wells except negative control. Two-fold dilutions of the preparations (antibiotics, nanoparticles coupled antibiotics, and gel-based nanoparticles and antibiotics) starting at 10,000 µg/mL (10 mg/mL) were poured into wells. OD values at 630nm were taken at each 4 h interval until 24 h of incubation.

### 4.8. Statistical Analysis

The data on the estimation of risk factors was analyzed using the chi-square and regression analysis, and the prevalence was calculated using a descriptive statistic employing the formula as mentioned below; a *t*-test was conducted for the comparison of means of two groups; and ANOVA with tukey test was applied to analyze the data of more than two groups. SPSS version 22 software was applied to analyze data assuming a 5% probability for declaration of significant findings.

$$Prevalence(\%)=\frac{No\;of\;positive\;sample}{Total\;no\;of\;samples\;tested}\times 100$$

## Figures and Tables

**Table 1:** Risk factor analysis of *S. aureus*-based endometritis.

Parameter	Categories	Chi-Square Analysis			Regression Analysis			
		Frequency	% Age	*p*-Value	ODD’s Ration	CI (95%) for Odds Ratio		*p*-Value
						Lower	Upper	
Calving history	Dystocia	12	40	<0.001	0.444	0.158	1.249	0.124
	Abortion	0	0		0	0	0	0
	Eutocia	18	60		-	-	-	-
Milk yield/day	10–20	4	13.33	<0.001	0.103	0.028	0.369	0.001
	21–30	18	60		0.615	0.155	2.450	0.491
	31–40	6	20		2.154	0.363	12.764	0.398
	41–50	2	6.67		-	-	-	-
Days in milk	1–100	18	60	<0.001	3.500	1.201	10.196	0.022
	101–200	9	30		13.500	3.333	54.673	0.001
	201–300	3	10		-	-	-	-
Parity	1–3	19	63.33	0.039	2.983	1.044	8.527	0.041
	4–6	11	36.67		-	-	-	-
Feeding regime	Silage + Concentrate	2	6.67	<0.001	56.00	10.327	303.682	<0.001
	Silage + Hay + Concentrate	4	13.33		26.00	6.532	103.498	0.001

**Table 2:** Risk factor analysis for endometritis against *E. coli*.

		*E. coli*			Regression Analysis			
Parameter	Categories	Frequency	% Age	*p*-Value	ODD’s Ration	Lower	Upper	*p*-Value
Calving history	Dystocia	12	42.86	<0.001	1.538	0.536	4.416	0.423
	Abortion	1	3.57		31.154	3.704	262.059	0.002
	Eutocia	15	53.57		-	-	-	-
Milk yield/day	10–20	5	17.86	0.002	1.533	0.422	5.577	0.516
	21–30	14	50		0.33	0.108	1.034	0.057
	31–40	7	25		1.000	0.298	3.353	1.000
	41–50	2	7.14		-	-	-	-
Days in milk	1–100	16	57.14	0.001	0.09	0.022	0.369	0.001
	101–200	9	32.14		0.253	0.060	1.065	0.061
	201–300	3	10.72		-	-	-	-
Parity	1–3	19	67.86	<0.001	4.457	1.452	13.681	0.009
	4–6	9	32.14		0.106	-	-	-
Feeding regime	Silage + Concentrate	4	14.29	0.593	78.000	13.078	465.196	0.000
	Silage + Hay + Concentrate	4	14.28		36.000	8.057	160.849	<0.001
	Silage + Concentrate + Fresh fodder				-	-	-	-
	Combination	15	53.57		0.185			0.066

**Table 3:** Antibiotic susceptibility profile of *S. aureus* and *E. coli* against different antibiotics.

Antibiotics	Potency	*S. aureus*			*E. coli*		
		Resistant(R) %	Intermediate(I) %	Sensitive(S) %	Resistant(R) %	Intermediate(I) %	Sensitive(S) %
Fusidic acid	10 µg	60	20	20	60	20	20
Enrofloxacin	10 µg	20	20	60	10	10	80
Ciprofloxacin	5 µg	10	20	70	10	20	70
Levofloxacin	5 µg	30	30	40	30	30	40
Chloramphenicol	30 µg	20	30	50	50	30	20
Vancomycin	30 µg	10	20	70	10	20	70
Gentamicin	10 µg	10	10	80	60	10	30
Linezolid	30 µg	30	10	60	30	10	60
Cefoxitin	30 µg	50	10	40	50	10	40

**Table 4:** Toxicity evaluation of magnesium oxide with different preparations.

500 Preparation Name	Concentration Stock	No. of Snails Tested	Mortality Until Day 1		Mortality Until Day 3		Mortality Until Day 5	
			Ratio	%	Ratio	%	Ratio	%
MgO (Group 1)	10 mg/mL	5	1/5	20	3/5	60	4/5	80
	1 mg/mL	5	1/5	20	2/5	40	3/5	60
	0.1 mg/mL	5	0/5	0	1/5	20	2/5	40
	0.01 mg/mL	5	0/5	0	1/5	20	1/5	20
Control (Group 2)	-	10	0	0	0	0	2/10	20

## Data Availability

All the data is available in the manuscript.
